# Cross-sectional comparison of the association between three different insulin resistance surrogates and frailty: NHANES 1999-2018

**DOI:** 10.3389/fendo.2024.1439326

**Published:** 2024-08-23

**Authors:** Tianjie Lai, Fenglei Guan, Yunxian Chen, Konghe Hu

**Affiliations:** ^1^ Department of Spine Surgery, The Affiliated Yuebei People’s Hospital of Shantou University Medical College, Shaoguan, Guangdong, China; ^2^ Department of Cardiology, The Affiliated Yuebei People’s Hospital of Shantou University Medical College, Shaoguan, Guangdong, China

**Keywords:** insulin resistance surrogates, frailty, TyG, METS-IR, HOMA-IR, NHANES

## Abstract

**Background:**

The correlation between various insulin resistance surrogates and frailty remains under investigation in the scientific community.

**Methods:**

Data from NHANES (1999-2018) were used. We utilized weighted binary logistic regression, trend tests, RCS analysis, and subgroup analysis to comprehensively assess the link between METS-IR, HOMA-IR, and TyG, and frailty risk.

**Results:**

The results revealed a significant positive association between high levels of METS-IR, HOMA-IR, and TyG with the risk of frailty in all models. Notably, in model 4, the highest quintile of METS-IR showed the strongest link (OR: 2.960, 95% CI: 2.219-3.949), with HOMA-IR (OR: 2.522, 95% CI: 1.927-3.301) following closely behind. Trend tests revealed a positive trend between METS-IR, HOMA-IR, and TyG with the risk of frailty (P for trend < 0.05). RCS analysis showed a linear relationship between METS-IR and the risk of frailty (P for nonlinearity > 0.05). In contrast, HOMA-IR and TyG exhibited a U-shaped nonlinear relationship (P for nonlinearity < 0.05).

**Conclusion:**

The research identified a linear association between METS-IR and frailty risk, whereas HOMA-IR and TyG displayed a U-shaped, nonlinear relationship pattern with the risk of frailty. Among the varying levels examined, the linkage between METS-IR and frailty was most pronounced in the top quintile.

## 1 Introduction

The term “frailty” was once considered synonymous with disability, frequently linked with advanced age and various underlying conditions. However, with the progression of research, an increasing number of scholars recognize that frailty is a biological syndrome resulting from the decline of multiple physiological systems. It indicates a decrease in the body’s normal physiological reserves and resistance to stressors, causing individuals to face worse-than-expected outcomes compared to those with normal physical conditions (1, 2). Although frailty is typically defined as a common syndrome in the elderly, recent studies have increasingly shown that middle-aged and even young adults may also exhibit similar frailty symptoms (3). These symptoms may be associated with various background factors such as lifestyle, socioeconomic status, and health behaviors (4). A community study showed that the prevalence of frailty in the community is about 6.9%, and this rate increases with age (1). Frailty develops relatively slowly, particularly in its early stages (5). However, the definition of frailty is considered to also apply to younger individuals with physical frailty, but most current intervention trials mainly involve older populations, thus frailty in younger individuals has not received adequate attention (6). Given the negative impact of functional decline associated with frailty, early identification and prevention of frailty progression are essential (7). However, there is no clear gold standard for diagnosing frailty. Its common characteristics include unintentional weight loss, impaired physical function, feelings of weakness, fatigue, and low levels of physical activity levels. Currently, tools for assessing frailty include the Fried Frailty Phenotype (FFP) (1), the Frailty Index (FI) (8), and the Frailty Questionnaire (FRAIL) (9). Among these tools, the FI has widespread application value in assessing the characteristics of frail populations. By comprehensively considering multiple physiological and cognitive indicators, the FI can more fully reflect an individual’s level of frailty (10).

Insulin resistance (IR) refers to the decreased or impaired insulin sensitivity of target organs or tissues, resulting in a reduced efficiency of insulin-stimulated glucose utilization. IR frequently causes vascular changes and impairs organ functionality, and is regarded as a fundamental basis for many diseases. RI and diabetes are exhibiting a trend of early onset, and recognizing insulin resistance can aid in identifying early declines in bodily capabilities. However, the euglycemic-hyperinsulinemic clamp, the gold standard for evaluating IR, is not widely utilized because of its complexity and invasive nature. Consequently, numerous alternative indicators of IR have been developed, such as the Triglyceride-Glucose Index (TyG), Metabolic Score for Insulin Resistance (METS-IR), and Homeostatic Model Assessment of Insulin Resistance (HOMA-IR). Most of these indicators reflect the body’s glucose metabolism by blood glucose and lipid levels. Due to their ease of acquisition and simple calculation, they have shown certain advantages in studies of diseases such as coronary heart disease and hypertension (11). However, the relationship between insulin resistance, which is a foundation for cardiovascular diseases and aging, and frailty has not been widely studied. We aim to explore the association between insulin resistance and frailty, as defined by the FI index, using different IR substitutes in the general population and to compare their predictive abilities.

As far as we know, we are the first to conduct a cross-sectional comparison of the relationship between different insulin resistance surrogates and frailty. In summary, we used data from NHANES (National Health and Nutrition Examination Survey) 1999-2018 to study the association between different insulin substitutes and the occurrence of frailty. This may provide new insights for clinical treatment and prevention.

## 2 Materials and methods

### 2.1 Data source and study population

**Figure 1:**
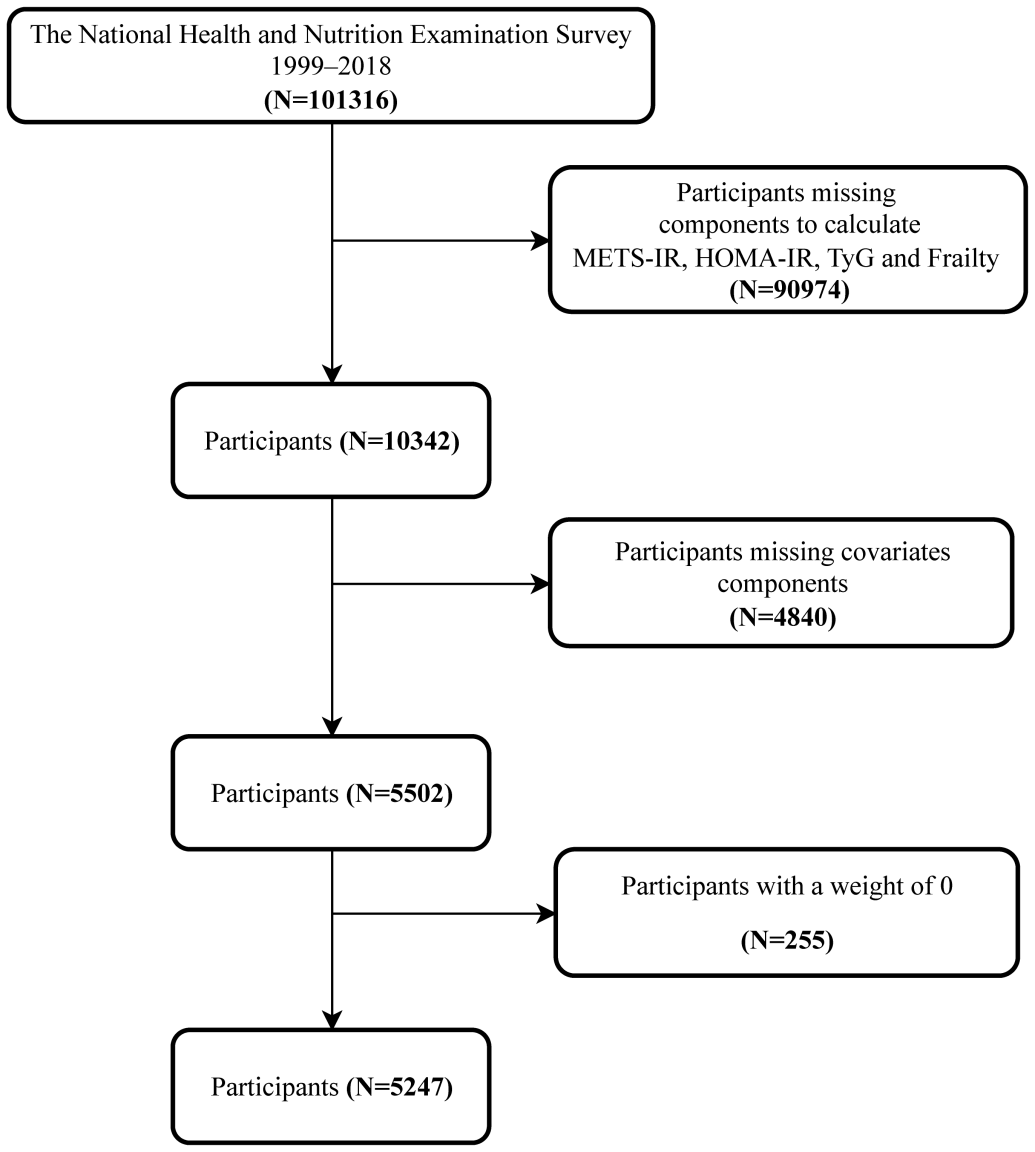
Flowchart of the participants’ selection.

This study was based on data from the NHANES 1999-2018 and included 5247 participants aged 20 years and older. NHANES is a large, multistage, probability sampling national representative health survey conducted by the National Center for Health Statistics (NCHS), aimed at assessing the health and nutritional status of the non-institutionalized population in the United States. The participant selection process is shown in **Figure 1**. Exclusion criteria included: 1. Missing modules required for calculating METS-IR, HOMA-IR, TyG, and frailty scores; 2. Participants missing other covariate modules; 3. Remove participants with a weight of 0.

### 2.2 Frailty assessment

In our study, the construction of FI follows a series of specific criteria proposed by Searle (12). The FI index is based on the accumulation of defects involving health defects that are not only prevalent but also typically worsen with age, covering multiple physiological and psychological systems of individuals, and at least 80% of health defects are recorded in participants. FI is a continuous scoring system that calculates the number of acquired deficits in multiple life domains of individuals and divides it by the total number of potential deficits to obtain. These deficits include cognitive function, dependency on daily activities, depressive symptoms, comorbidities, hospital utilization rate, overall health status, physical function, anthropometry (e.g., BMI), and laboratory test results. Each deficit is scored based on its potential impact on individual function, typically ranging from 0 to 1. By referring to previous studies (13, 14), based on the score of FI, we set a threshold of 0.21, defining individuals as frail when their score reaches or exceeds this value, and non-frail when it is below this value. (More details are shown in **Supplementary Table 1**).

### 2.3 Explanatory variables

The surrogate indexes of IR in this study include HOMA-IR, TyG, and METS-IR. These IR surrogates are derived from blood samples taken after an overnight fast, collecting data on total cholesterol, high-density lipoprotein (HDL), low-density lipoprotein (LDL), triglycerides, fasting blood glucose, and insulin. The specific calculation formulas are as follows:


$$\text{HOMA-IR}=\frac{\text{fasting glucose (mmol/L)}\times \text{fasting insulin (μU/mL)}}{22.5}$$

$$\text{TyG}=\ln\left(\frac{\text{fasting triglycerides (mg/dL)}\times \text{fasting glucose (mg/dL)}}{2}\right)$$

$$\text{METS-IR}=\frac{\ln\left(2\times \text{fasting glucose (mg/dL)}+\text{fasting triglycerides (mg/dL)}\right)\times {\text{body mass index (kg/m}}^{2})}{\ln\left(\text{high-density lipoprotein cholesterol (mg/dL)}\right)}$$

### 2.4 Definitions of covariates

This study included multiple covariates to analyze their potential effects on the main variables comprehensively. These covariates include age, sex, race (Non-Hispanic Black, Non-Hispanic White, Other/Multiracial, Mexican American, Other Hispanic), alcohol use (Never, Former, Mild, Moderate, Heavy), diet quality, total energy intake, physical activity, smoking status (Never, Former, Now), income level (Poor and Not poor), marital status (Never married, Married/Living with Partner, Widowed/Divorced/Separated), and education attainment (Less than 9th grade, 9-11th Grade, High school grad/GED, Some college or AA degree, College graduate or above).

### 2.5 Statistical analysis

In this study, we considered the complex, multistage sampling design used by NHANES. Following official guidelines, we incorporated sample weights, stratification, and clustering into all statistical analyses, adhering to NHANES’s analysis and reporting recommendations. Baseline characteristics of participants were classified according to their frailty status. For continuous variables, the means and 95% confidence intervals (CIs) were reported. For categorical variables, percentages and 95% CIs were used. Differences between groups were analyzed using the Wilcoxon rank-sum test for continuous variables and the chi-square test with Rao & Scott’s second-order correction for categorical variables, which better handles the complexities of the sample design.

We used weighted logistic regression analysis to evaluate the linear association between METS-IR, HOMA-IR, TyG, and frailty, expressing the strength of associations with odds ratios (ORs) and 95% CIs. We constructed four models: Model 1 was unadjusted, Model 2 was adjusted for age, sex, and race (key factors in NHANES’s design), and Model 3 was adjusted for sex, smoking status, and education level. Variables included in Model 3 were if their inclusion or exclusion caused a change in the regression coefficient for the primary variable (X) by more than 10%. Model 4 was adjusted for all considered covariates, including age, sex, race, alcohol use, education level, smoking status, diet quality, total energy intake, physical activity, income level, and marital status.

Additionally, METS-IR, HOMA-IR, and TyG were divided into quintiles. Weighted logistic regression was used to assess the association between each quintile and frailty, with trend tests conducted to evaluate consistent changes across index levels. We further analyzed potential nonlinear relationships between these IR surrogates and frailty using restricted cubic spline analysis, adjusting for all covariates and evaluating nonlinear relationships through likelihood ratio tests.

We performed subgroup analyses to explore potential factors influencing the associations between METS-IR, HOMA-IR, TyG, and frailty, considering age, sex, race, alcohol use, education level, smoking status, income level, and marital status. A two-tailed p-value < 0.05 was deemed statistically significant, and all analyses were conducted using R software version 4.3.1.

### 2.6 Ethics approval and consent to participate

This study adhered to the ethical standards of the Helsinki Declaration and its amendments or equivalent standards. The data used was sourced from the NHANES survey, which was approved by the NCHS Ethics Review Board. As this research involved secondary analysis of the NHANES database and complied with STROBE guidelines, no additional ethical approval was required. For detailed NHANES methodology and ethical standards, visit the CDC and NCHS websites: https://www.cdc.gov/nchs/nhanes/irba98.htm.

## 3 Results

### 3.1 General characteristics of participants

As shown in **Table 1**, our study included 5,247 participants. Comparing frail and non-frail groups revealed no significant difference in mean age (both groups averaged 59 years). Frail individuals were more likely to be female, have poorer diet quality, lower energy intake and physical activity levels, higher prevalence of alcohol use and smoking, lower income and education levels, and were more likely to be widowed, divorced, or separated.

**Table 1:** General characteristics of participants.

	Frailty (N= 5247)		
Characteristic	No, N = 3874^12^	Yes, N = 1373^12^	P Value^3^
**Age, years**	59 [58, 59]	59 [58, 60]	0.6
**Diet quality (HEI-2015)**	53 [52, 53]	49 [48, 50]	**<0.001**
**Total energy, kcal**	2,117 [2,076, 2,158]	1,939 [1,877, 2,001]	**<0.001**
**Physical activity (MET × min/week)**	3,114 [2,860, 3,369]	2,556 [2,266, 2,845]	**<0.001**
**Sex, %**			**<0.001**
Male	52 [50, 54]	40 [37, 44]	
Female	48 [46, 50]	60 [56, 63]	
**Race, %**			**<0.001**
Non-hispanic black	7.1 [6.1, 8.4]	12 [9.9, 14]	
Non-hispanic white	81 [78, 83]	73 [69, 77]	
Other/multiracial	5.1 [4.2, 6.1]	5.9 [4.4, 7.9]	
Mexican American	3.9 [3.2, 4.7]	4.1 [3.2, 5.2]	
Other hispanic	3.3 [2.7, 4.0]	5.0 [3.5, 7.3]	
**Alcohol use, %**			**<0.001**
Never	11 [9.4, 12]	9.9 [8.1, 12]	
Former	16 [15, 17]	27 [23, 30]	
Mild	45 [43, 48]	34 [30, 38]	
Moderate	14 [13, 16]	12 [10, 15]	
Heavy	14 [12, 15]	17 [14, 20]	
**Income level, %**			**<0.001**
Poor	9.3 [8.0, 11]	24 [21, 28]	
Not poor	91 [89, 92]	76 [72, 79]	
**Smoke status, %**			**<0.001**
Never	47 [45, 49]	36 [33, 39]	
Former	36 [34, 38]	35 [32, 39]	
Now	17 [16, 19]	29 [26, 32]	
**Education attainment, %**			**<0.001**
Less than 9th grade	4.2 [3.6, 4.8]	8.0 [6.5, 9.9]	
9-11th Grade	9.1 [8.0, 10]	15 [13, 18]	
High school grad/GED	24 [22, 27]	31 [28, 34]	
Some college or AA degree	31 [29, 34]	31 [28, 35]	
College graduate or above	31 [28, 34]	14 [12, 17]	
**Marital status, %**			**<0.001**
Never married	10 [8.9, 12]	8.3 [6.5, 11]	
Married/Living with Partner	69 [66, 71]	58 [54, 62]	
Widowed/ Divorced/ Separated	21 [20, 23]	33 [30, 37]	

^1^Mean; %.

^2^CI, Confidence Interval.

^3^Wilcoxon rank-sum test for complex survey samples; chi-squared test with Rao & Scott’s second-order correction.

P value <0.05 is considered to have statistical significance and has been highlighted in bold.

### 3.2 Association between different IR surrogates and frailty

Using weighted multivariable logistic regression, we assessed the relationship between METS-IR, HOMA-IR, TyG, and frailty risk, as presented in **Table 2**. Both METS-IR and TyG showed significant positive associations with frailty risk across all models. In Model 4, which adjusted for all covariates, the Odds Ratio (OR) for TyG was 1.413 (95% CI: 1.202, 1.661), and for METS-IR was 1.036 (95% CI: 1.028, 1.044). HOMA-IR showed significant positive associations in Models 1 and 2, but these were not statistically significant in Models 3 and 4 after adjusting for additional covariates.

**Table 2:** Association between different IR surrogates and frailty.

	Model 1			Model 2			Model 3			Model 4		
Characteristic^1^	OR^2^	95% CI^2^	p-value	OR^2^	95% CI^2^	p-value	OR^2^	95% CI^2^	p-value	OR^2^	95% CI^2^	p-value
METS-IR	1.035	1.028, 1.042	**<0.001**	1.037	1.030, 1.044	**<0.001**	1.036	1.029, 1.043	**<0.001**	1.036	1.028, 1.044	**<0.001**
HOMA-IR	1.047	1.001, 1.096	**0.046**	1.050	1.000, 1.101	**0.050**	1.050	0.9991, 1.104	0.054	1.044	0.9895, 1.102	0.11
TyG	1.467	1.279, 1.684	**<0.001**	1.574	1.358, 1.824	**<0.001**	1.409	1.218, 1.630	**<0.001**	1.413	1.202, 1.661	**<0.001**
METS-IR Q5												
Q1	—	—		—	—		—	—		—	—	
Q2	0.9902	0.7450, 1.316	>0.9	1.057	0.7801, 1.432	0.7	1.060	0.7864, 1.428	0.7	1.032	0.7613, 1.400	0.8
Q3	1.334	1.031, 1.727	**0.028**	1.432	1.095, 1.873	**0.009**	1.453	1.105, 1.909	**0.008**	1.411	1.066, 1.867	**0.017**
Q4	1.827	1.371, 2.435	**<0.001**	2.066	1.525, 2.799	**<0.001**	1.928	1.423, 2.613	**<0.001**	1.865	1.361, 2.556	**<0.001**
Q5	2.830	2.210, 3.624	**<0.001**	3.105	2.392, 4.030	**<0.001**	2.982	2.294, 3.874	**<0.001**	2.960	2.219, 3.949	**<0.001**
HOMA-IR Q5												
Q1	—	—		—	—		—	—		—	—	
Q2	0.8631	0.6562, 1.135	0.3	0.8732	0.6582, 1.158	0.3	0.8827	0.6640, 1.173	0.4	0.9018	0.6781, 1.200	0.5
Q3	1.033	0.8056, 1.325	0.8	1.050	0.8139, 1.355	0.7	1.080	0.8348, 1.396	0.6	1.055	0.8155, 1.364	0.7
Q4	1.360	1.057, 1.751	**0.017**	1.388	1.079, 1.785	**0.011**	1.449	1.126, 1.863	**0.004**	1.431	1.098, 1.864	**0.008**
Q5	2.395	1.887, 3.039	**<0.001**	2.517	1.970, 3.215	**<0.001**	2.587	2.005, 3.338	**<0.001**	2.522	1.927, 3.301	**<0.001**
TyG Q5												
Q1	—	—		—	—		—	—		—	—	
Q2	1.086	0.7911, 1.491	0.6	1.110	0.8136, 1.514	0.5	1.029	0.7605, 1.392	0.9	0.9539	0.7096, 1.282	0.8
Q3	1.378	1.002, 1.896	**0.049**	1.442	1.041, 1.998	**0.028**	1.318	0.9571, 1.815	0.090	1.237	0.8996, 1.701	0.2
Q4	1.428	1.084, 1.881	**0.012**	1.521	1.152, 2.007	**0.003**	1.358	1.026, 1.799	**0.033**	1.280	0.9632, 1.701	0.088
Q5	2.193	1.686, 2.852	**<0.001**	2.485	1.892, 3.265	**<0.001**	1.989	1.513, 2.615	**<0.001**	1.969	1.483, 2.614	**<0.001**

^1^Models: Model 1: Not adjusted; Model 2: Adjusted Age, Sex, Race; Model 3: Adjusted Sex, Smoke status, Education attainment; Model 4: Adjusted Age, Sex, Race, Alcohol use, Income level, Smoke status, Education attainment, MET, HEI, Total energy, Marital status.

^2^OR, Odds Ratio; CI, Confidence Interval. P value <0.05 is considered to have statistical significance and has been highlighted in bold.

We divided these indices into quintiles to better compare the associations between METS-IR, HOMA-IR, TyG, and frailty. In Model 4, the highest quintile (Q5) for METS-IR had an OR of 2.960 (95% CI: 2.219, 3.949); for HOMA-IR, the OR was 2.522 (95% CI: 1.927, 3.301). Notably, HOMA-IR and TyG in the second quintile (Q2) showed a non-significant negative association with frailty, suggesting a potential nonlinear relationship.

### 3.3 Trend tests of association between different IR surrogates and frailty

**Figure 2:**
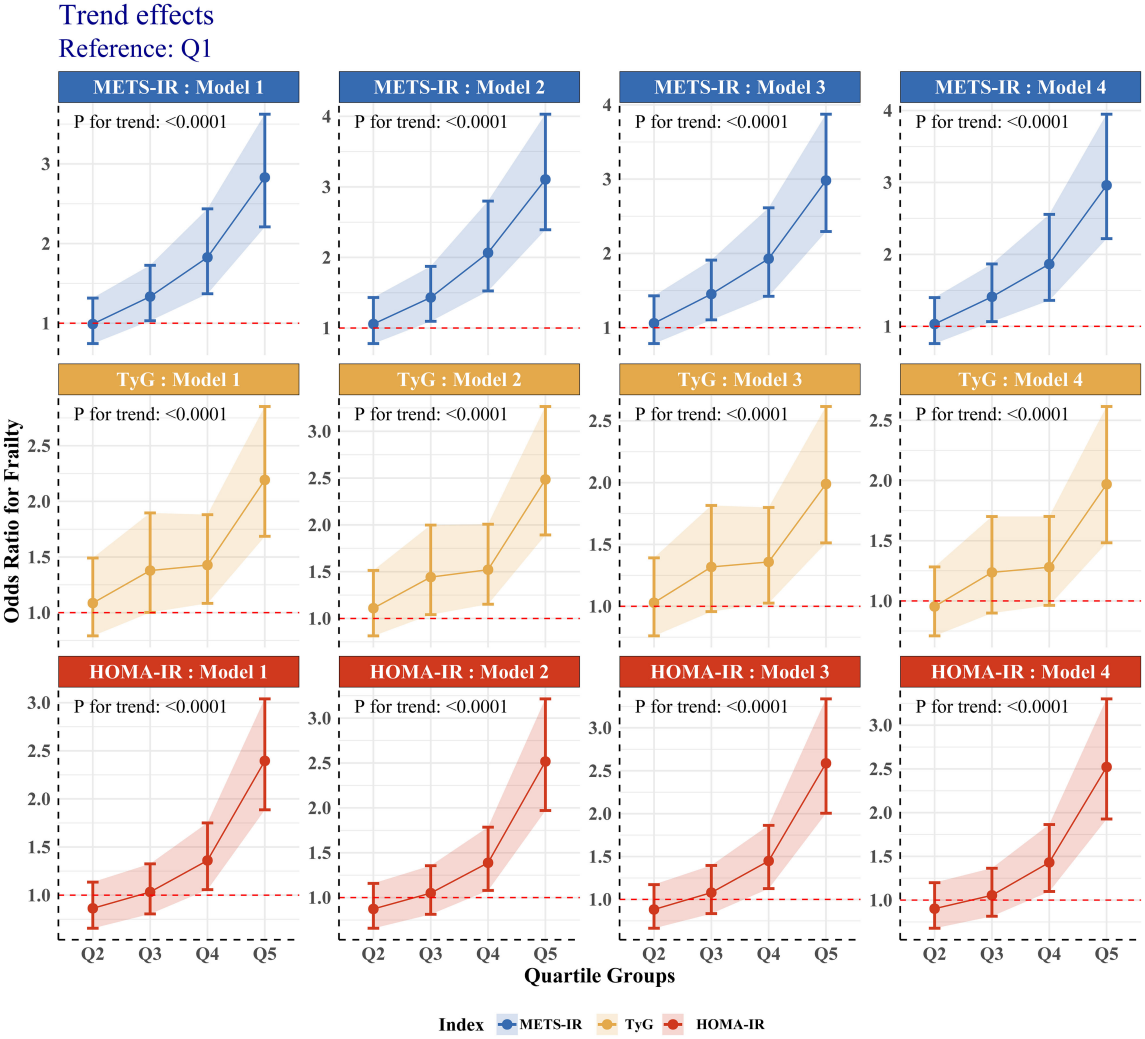
Trend tests of association between different IR surrogates and frailty.

We divided METS-IR, HOMA-IR, and TyG into quintiles and conducted trend tests. Results in **Figure 2** show a clear trend effect between these IR surrogates and frailty (P for trend < 0.05). Compared to the Q1 group, the risk of frailty rises as the levels of these indices increase.

### 3.4 Restricted Cubic Spline analysis of the association between different IR surrogates and frailty

**Figure 3:**
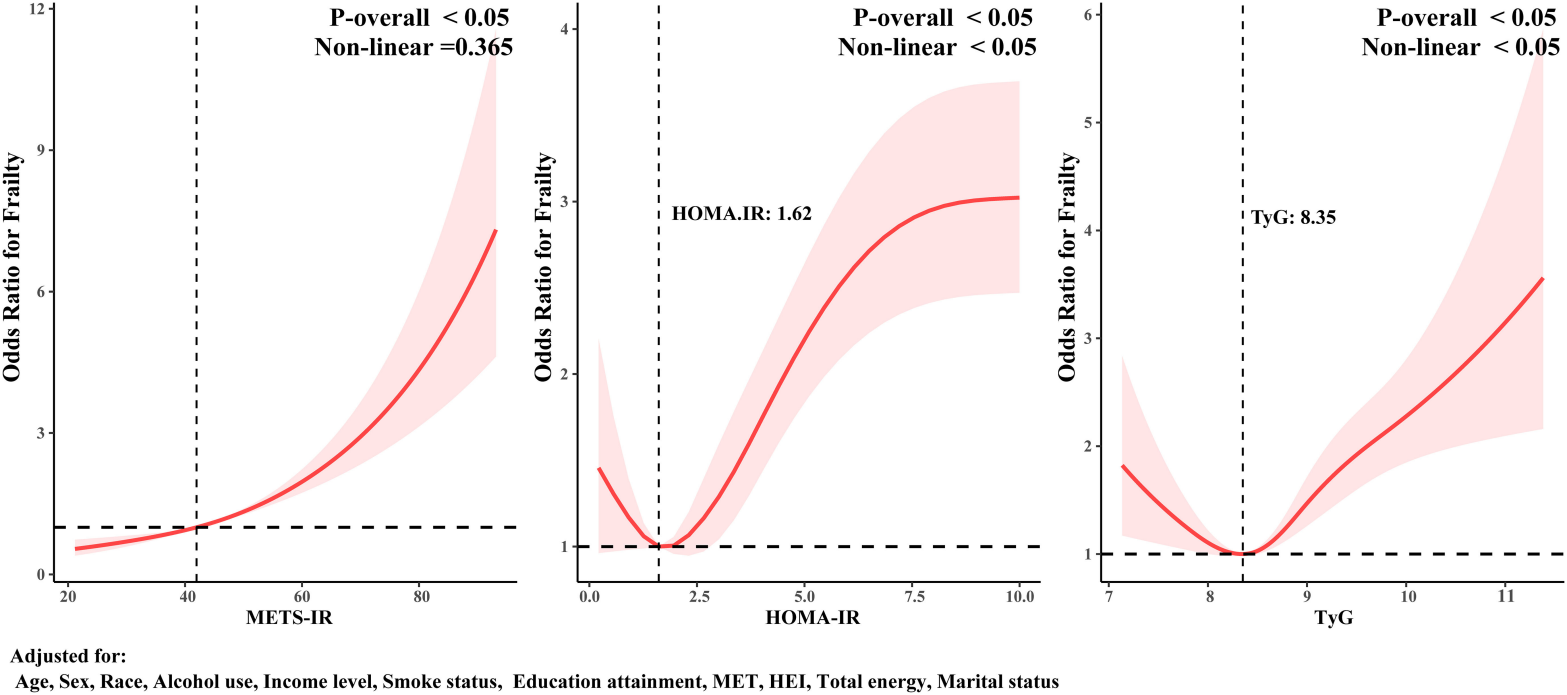
RCS analysis of the association between different IR surrogates and frailty.

We performed a weighted RCS analysis with adjustments for all relevant covariates to better fit the relationship between METS-IR, HOMA-IR, TyG, and frailty risk. The results in **Figure 3** indicate that the relationship between METS-IR and frailty risk is likely linear (P-Nonlinear > 0.05). On the other hand, HOMA-IR and TyG have U-shaped relationships with frailty risk (P-Nonlinear < 0.05). As the levels of HOMA-IR and TyG increase, frailty risk first decreases, but after surpassing a threshold, the risk increases. This is consistent with the findings presented in **Table 2**; **Figure 2**.

### 3.5 Subgroup analysis of the relationship between different IR surrogates and frailty

**Figure 4:**
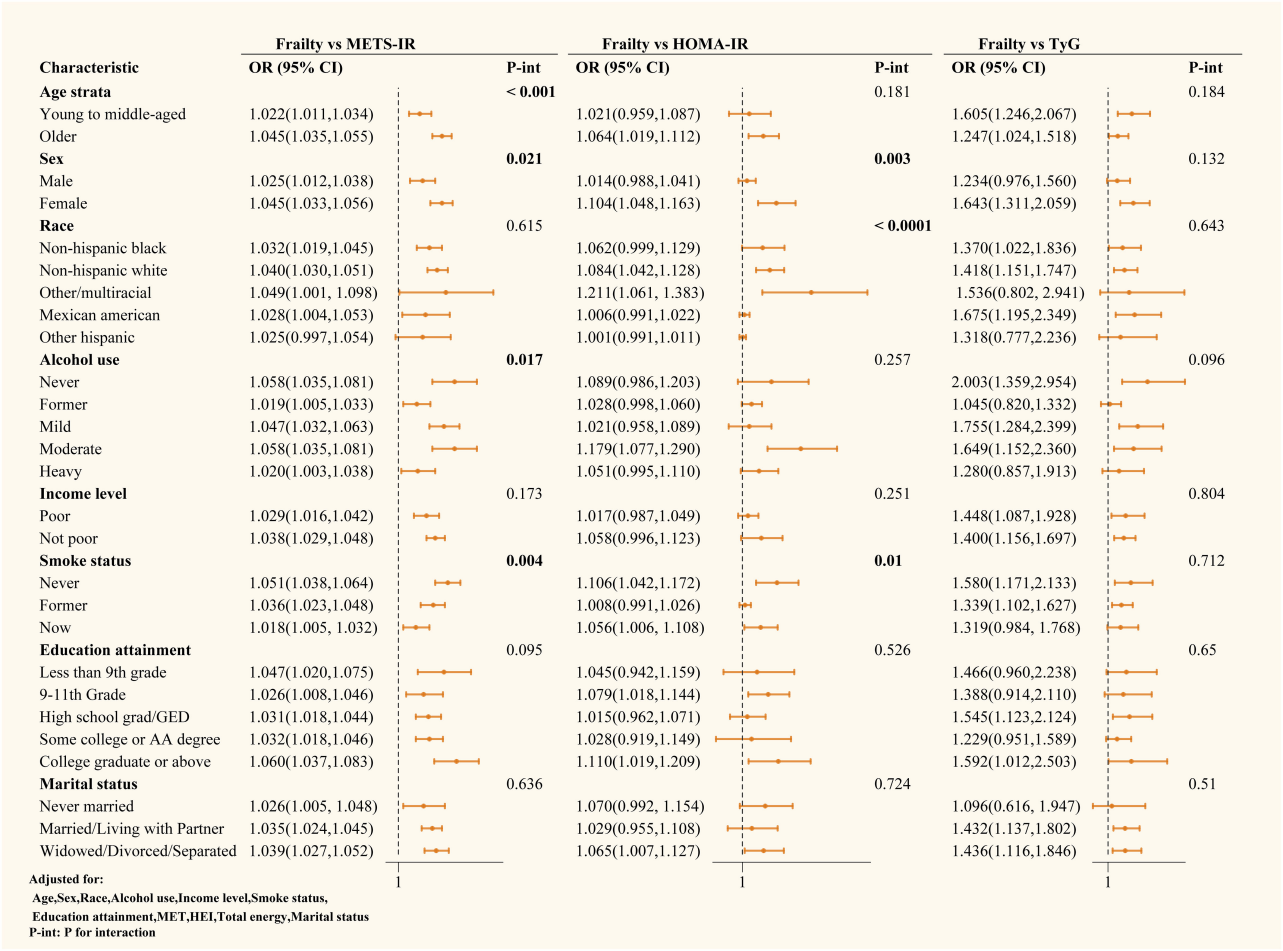
Subgroup analysis of the relationship between different IR surrogates and frailty.

Subgroup analyses explored the impact of different variables on the associations between METS-IR, HOMA-IR, TyG, and frailty risk. Detailed results are presented in **Figure 4**. TyG’s association with frailty was robust across all subgroups (P for interaction > 0.05). In contrast, METS-IR’s association with frailty was influenced by age, sex, alcohol use, and smoking status (P for interaction < 0.05), being more significant in older adults, females, and non-smokers. The HOMA-IR and frailty association was affected by sex, race, and smoking status, showing more significant associations in females and non-smokers.

## 4 Discussion

This study explored the relationship between different IR surrogates (METS-IR, HOMA-IR, and TyG) and frailty risk among 5,247 individuals from U.S. communities. The study revealed that despite the similar average age between the frail and non-frail groups, there were significant differences in gender distribution, dietary habits, lifestyle, and socioeconomic status in the frail group. Through weighted multivariate logistic regression analysis, high METS-IR, HOMA-IR, and TyG levels were significantly associated with frailty risk, with METS-IR in the highest quartile showing the strongest association with frailty.

RCS observed a possible linear relationship between METS-IR and frailty risk. At the same time, HOMA-IR and TyG showed a U-shaped nonlinear relationship with frailty risk. This finding suggests that different measures of insulin resistance might have distinct mechanisms in predicting frailty risk. Additionally, subgroup analysis further revealed that factors such as age, gender, race, alcohol use, and smoking status might influence these associations. Specifically, the association between METS-IR and frailty was more pronounced in older adults, women, and non-smokers. In contrast, HOMA-IR and frailty were more significant in women and non-smokers.

Numerous previous studies have noted the relationship between insulin resistance and frailty. A cross-sectional study involving 3,141 community-dwelling adults aged 69 to 74 years showed that HOMA-IR levels were positively associated with frailty risk; each standard deviation increase in HOMA-IR was associated with a 15% increase in frailty risk, and this positive association was also observed in pre-frailty (15). Muscle wasting and decreased muscle mass are considered characteristics of frailty. Research by Christine et al. discovered that with increased HOMA-IR, weight, total lean mass, and appendicular lean mass decreased (16). Another study involving 2,403 Korean participants aged 70-84 found that men with high insulin resistance assessed by HOMA-IR had a significantly higher prevalence of sarcopenia compared to insulin-sensitive individuals (17). Other studies have also explained that increasing muscle mass can prevent IR. Ghachem et al. found that lean body mass is an independent predictor of IR, and increasing muscle mass can improve IR (18). Ahn et al. studied IR based on the TyG in the Korean population and found that groups with a lower skeletal muscle mass index had higher IR levels (19). Lower grip strength was also found to be associated with a high TyG, potentially related to the inactivation of insulin receptor substrates and loss of muscle mass (20). In a recent research, Yin Yuan et al. highlighted the relationship between the TyG and frailty, indicating that a long-term increase in the TyG is independently associated with an increased risk of frailty (7). This provides new evidence, besides HOMA-IR, directly reflecting the association between insulin resistance and frailty.

To further understand the association between different surrogate markers of insulin resistance and frailty, we adjusted for as many variables as possible in Model 4. The results showed that all different insulin resistance surrogates were correlated with frailty. Among them, METS-IR exhibited superior performance in predicting frailty risk when compared to TyG and HOMA-IR. One possible reason is that the METS-IR index takes into account not only fasting glucose and fasting triglycerides but also body mass index (BMI) and high-density lipoprotein cholesterol (HDL-C), making METS-IR more comprehensive in reflecting the complexity of insulin resistance. In contrast, HOMA-IR is based solely on fasting glucose and fasting insulin, while the TyG index considers only fasting triglycerides and fasting glucose. Several studies have found that indices including BMI-related factors often have higher predictive value, such as METS-IR and TyG-BMI (21). Consequently, METS-IR might more accurately capture the multidimensional features of metabolic syndrome, making it more effective in predicting the risk of frailty. This conclusion aligns with findings from other studies. For example, in evaluating the presence and severity of Coronary Artery Disease (CAD) (22), hyperuricemia, and the prevalence of periodontitis (23, 24), METS-IR demonstrated better predictive value among different non-insulin-based IR indexes23.

However, the mechanism linking IR to frailty is still unclear. Some studies suggest muscle metabolism is the main bridge between IR and aging. Most studies have observed that people with insulin resistance have lower lean body mass, higher fat mass, and often a decline in muscle mass (16, 20). This may be related to the weight loss and muscle strength decline commonly seen in frail populations (25). This may be associated with IR impairing muscle metabolism in frail individuals, which exacerbates the decline in lean body mass and muscle mass. Linda et al. observed improvements in muscle perfusion and vascular insulin resistance in diabetic patients treated with Empagliflozin (26). Another crossover study involving 13 young subjects found that interrupting sedentary behavior can lower postprandial glucose levels (27). The mechanism may involve muscle-releasing growth factors during physical activity, stimulating muscle growth, and improving insulin sensitivity. One can assume that reduced activity, one of the main features of frailty, is an essential contributor to insulin resistance.

IR is also thought to have a bridging role in the development of inflammation and frailty. A cross-sectional study analyzing 529 adolescents aged 12 to 18 found that HOMA-IR was positively correlated with inflammation scores and negatively correlated with low muscle health (28). Unhealthy adolescents with high inflammatory biomarkers exhibited high HOMA-IR. Additionally, older adults have a reduced ability to process specific components of carbohydrates, which may contribute to oxidative stress or inflammatory responses in the body (29). The mechanisms involved may be related to the fact that insulin resistance increases oxidative stress and inflammatory reactions in the body. Linda et al. (26) and Mone et al. (30, 31) have had success in improving insulin resistance. They found that by using the SGLT2 (sodium-dependent glucose transporters 2) inhibitor engeletin served to improve endothelial function and reduce mitochondrial oxidative stress in frail patients, epigenetically improving cognitive function and boosting muscle performance. In contrast, studies that attempted to use aspirin to simply inhibit inflammatory effects were observed to have no relief from the onset of frailty (32).

Given the points above, we boldly speculate that reduced activity due to multiple factors may be the initiating factor for frailty, a change that in turn triggers increased insulin resistance. Insulin resistance weakens muscle metabolism and may lead to adverse effects such as cognitive decline and oxidative stress, creating a vicious cycle that significantly accelerates the process of frailty. Thus, IR plays a central role as an intermediary in this process, and it is closely linked to the precursors of frailty, such as reduced activity, muscle mass, and cognitive decline (33, 34), which are common pathologic features of frail populations.

Finally, we are the first to conduct a cross-sectional comparison of the relationship between different insulin resistance surrogates and frailty. Our research supports that improving insulin resistance can alleviate frailty. Some prospective studies have also found that insulin sensitizers can mitigate muscle loss in diabetic populations (35). Randomized trials have also found that metformin can somewhat reduce the risk of frailty in diabetic and prediabetic individuals (36–38). Moreover, increasing muscle mass is believed to be an early preventive measure against insulin resistance and type 2 diabetes (39). Therefore, early physical activity interventions are a cost-effective approach to reducing insulin resistance and the risk of frailty (5).

## 5 Strengths and limitations

The NHANES database offers a large sample size and a sampling design that more accurately reflects the frail population in U.S. communities. We accounted for various factors, such as age, gender, and comorbidities, in our analysis to reduce potential bias and error, thereby enhancing the robustness of our main findings. Furthermore, in clinical practice, it is challenging to gather data on young patients, leading to a lack of focus on this group in current clinical research. On the other hand, public databases collect data from the community, effectively bridging this gap, and this is considered one of the strengths of the NHANES public database. Our study found an association between insulin resistance and frailty, with METS-IR outperforming TyG and HOMA-IR as a predictor. However, like other cross-sectional studies, we cannot establish a causal relationship between insulin resistance and frailty. Thus, our results should be interpreted cautiously, and this methodological constraint may limit our conclusions.

Additionally, despite the broad representativeness of the NHANES database, there may still be some potential biases. Other important variables or factors, such as genetics, environment, lifestyle, regional populations, and cultural backgrounds, may not have been considered. These missing variables could influence the results. Despite these limitations, our findings remain reliable and could provide valuable scientific evidence for public health strategies and chronic disease management.

## 6 Conclusions

The research identified a linear association between METS-IR and frailty risk, whereas HOMA-IR and TyG displayed a U-shaped, nonlinear relationship pattern with the risk of frailty. Among the varying levels examined, the linkage between METS-IR and frailty was most pronounced in the top quintile. In summary, higher insulin resistance levels correlated with frailty.

## Data Availability

Publicly available datasets were analyzed in this study. This data can be found here: https://www.cdc.gov/nchs/nhanes.
