# Supplementary material for: Cross-sectional comparison of the association between three different insulin resistance surrogates and frailty: NHANES 1999-2018
Source: Front Endocrinol (Lausanne). 2024 Aug 23;15:1439326. doi: 10.3389/fendo.2024.1439326 (PMC11377286; doi:10.3389/fendo.2024.1439326)

**Supplementary Table 1: List of Variables in the 49-Item Frailty Index with Corresponding Scores**

| **Variable** | **Scoring** | |
| --- | --- | --- |
| **Comorbidities** |  | |
| 1.Arthritis | Yes=1, Suspect = 0.5 No=0 | |
| 2.Thyroid problems | Yes=1, Suspect = 0.5 No=0 | |
| 3.Chronic Bronchitis | Yes=1, Suspect = 0.5 No=0 | |
| 4.Cancer | Yes=1, Suspect = 0.5 No=0 | |
| 5.Congestive Heart Failure | Yes=1, Suspect = 0.5 No=0 | |
| 6.Coronary Heart Disease | Yes=1, Suspect = 0.5 No=0 | |
| 7.Angina | Yes=1, Suspect = 0.5 No=0 | |
| 8.Heart Attack | Yes=1, Suspect = 0.5 No=0 | |
| 9.Stroke | Yes=1, Suspect = 0.5 No=0 | |
| 10.Blood Pressure | Yes=1, Suspect = 0.5 No=0 | |
| 11.Diabetes | Yes=1, Suspect = 0.5 No=0 | |
| 12.weak/failing kidneys | Yes=1, Suspect = 0.5 No=0 | |
| 13.Urinary Leakage | Yes=1, Suspect = 0.5 No=0 | |
| **Dependence** |  | |
| 14.Standing up from armless chair | Difficulty=1, No Difficulty=0 | |
| 15.Getting in and out of bed difficulty | Difficulty=1, No Difficulty=0 | |
| 16.Using fork, knife, drinking from cup | Difficulty=1, No Difficulty=0 | |
| 17.Dressing yourself | Difficulty=1, No Difficulty=0 | |
| 18.Standing for long periods difficulty | Difficulty=1, No Difficulty=0 | |
| 19.Grasp/holding small objects | Difficulty=1, No Difficulty=0 | |
| 20.Attending social event | Difficulty=1, No Difficulty=0 | |
| 21.Push or pull large objects | Difficulty=1, No Difficulty=0 | |
| 22.Walking for a quarter mile difficulty | Difficulty=1, No Difficulty=0 | |
| 23.Walking up ten steps difficulty  24. Managing money  25. Stooping, crouching, kneeling  26. Lifting or carrying  27. House chore  28. Preparing meals | Difficulty=1, No Difficulty=0  Difficulty=1, No Difficulty=0  Difficulty=1, No Difficulty=0  Difficulty=1, No Difficulty=0  Difficulty=1, No Difficulty=0  Difficulty=1, No Difficulty=0 | |
| **Cognition** |  | |
| 29.Experience confusion/memory problems | Yes=1, No=0 | |
| **Hospital Utilization & Access to Care** |  | |
| 30.Self Rated Health | Fair, poor=1, Excellent, Very good, good=0 | |
| 31.Health now compared with 1 year ago | Worse=1, About the same, better =0 | |
| 32.Overnight hospital patient in last year | Yes=1, No=0 | |
| 33.Frequency of health care use during last year. | None=0, 1-5= 0,5, More than 5=1 | |
| 34.Number of Prescribed medications | None=0 , 1-4=0.5, Five and more= 1 | |
| **Physical performance and Anthropometry** |  | |
| 35.Body Mass Index | <18.5, ≥ 30 =1  25-<30 = 0.5  18.5-25 =0 | |
| 36.Handgrip strength | **Males:**  For BMI ≤ 24, GS ≤ 29  For BMI 24.1–28, GS ≤ 30  For BMI >28, GS ≤ 32 =1 | **Females:**  For BMI ≤ 23, GS ≤ 17  For BMI 23.1–26, GS ≤ 17.3  For BMI 26.1–29, GS ≤ 18  For BMI>29, GS ≤ 21 =1 |
| **Laboratory Data** |  |  |
| 37.Glycohemoglobin (%) | 0-5.7 % =0, >5.7% =1 | |
| 38.Red blood cell count (million cells/μL) | M: 4.7-6.1= 0, Other =1 | F: 4.2-5.4=0, Other =1 |
| 39.Hemoglobin (g/dL) | M: 13.5-18= 0, Other=1 | F: 12-16 =0, Other = 1 |
| 40.Red cell distribution width (%) | 11.6-14.6 = 0, Other =1 | |
| 41.Lymphocyte percent (%) | 20- 40 = 0, Other=1 | |
| 42.Segmented neutrophils percent (%) | 40-80 = 0, Other=1 | |
| **Depressive Symptoms** |  | |
| 43. Have little interest in doing things | Nearly every day=1, More than half the days= 0.66, Several days= 0.33, Not at all=0 | |
| 44. Feeling down, depressed, or hopeless | Nearly every day=1, More than half the days= 0.66, Several days= 0.33, Not at all=0 | |
| 45. Trouble sleeping or sleeping too much | Nearly every day=1, More than half the days= 0.66, Several days= 0.33, Not at all=0 | |
| 46. Feeling tired or having little energy | Nearly every day=1, More than half the days= 0.66, Several days= 0.33, Not at all=0 | |
| 47. Poor appetite or overeating | Nearly every day=1, More than half the days= 0.66, Several days= 0.33, Not at all=0 | |
| 48. Feeling bad about yourself | Nearly every day=1, More than half the days= 0.66, Several days= 0.33, Not at all=0 | |
| 49. trouble concentrating on things | Nearly every day=1, More than half the days= 0.66, Several days= 0.33, Not at all=0 | |

**Supplementary Table 2: Subgroup analysis of the relationship between different IR surrogates and Frailty**

| **Characteristic** | **OR (95% CI)** | **P for interaction** | **OR (95% CI)** | **P for interaction** | **OR (95% CI)** | **P for interaction** |
| --- | --- | --- | --- | --- | --- | --- |
| **Age strata** |  | **< 0.001** |  | 0.181 |  | 0.184 |
| Young to middle-aged | 1.022(1.011,1.034) |  | 1.021(0.959,1.087) |  | 1.605(1.246,2.067) |  |
| Older | 1.045(1.035,1.055) |  | 1.064(1.019,1.112) |  | 1.247(1.024,1.518) |  |
| **Sex** |  | **0.021** |  | **0.003** |  | 0.132 |
| Male | 1.025(1.012,1.038) |  | 1.014(0.988,1.041) |  | 1.234(0.976,1.560) |  |
| Female | 1.045(1.033,1.056) |  | 1.104(1.048,1.163) |  | 1.643(1.311,2.059) |  |
| **Race** |  | 0.615 |  | **< 0.0001** |  | 0.643 |
| Non-hispanic black | 1.032(1.019,1.045) |  | 1.062(0.999,1.129) |  | 1.370(1.022,1.836) |  |
| Non-hispanic white | 1.040(1.030,1.051) |  | 1.084(1.042,1.128) |  | 1.418(1.151,1.747) |  |
| Other/multiracial | 1.049(1.001, 1.098) |  | 1.211(1.061, 1.383) |  | 1.536(0.802, 2.941) |  |
| Mexican american | 1.028(1.004,1.053) |  | 1.006(0.991,1.022) |  | 1.675(1.195,2.349) |  |
| Other hispanic | 1.025(0.997,1.054) |  | 1.001(0.991,1.011) |  | 1.318(0.777,2.236) |  |
| **Alcohol** **use** |  | **0.017** |  | 0.257 |  | 0.096 |
| Never | 1.058(1.035,1.081) |  | 1.089(0.986,1.203) |  | 2.003(1.359,2.954) |  |
| Former | 1.019(1.005,1.033) |  | 1.028(0.998,1.060) |  | 1.045(0.820,1.332) |  |
| Mild | 1.047(1.032,1.063) |  | 1.021(0.958,1.089) |  | 1.755(1.284,2.399) |  |
| Moderate | 1.058(1.035,1.081) |  | 1.179(1.077,1.290) |  | 1.649(1.152,2.360) |  |
| Heavy | 1.020(1.003,1.038) |  | 1.051(0.995,1.110) |  | 1.280(0.857,1.913) |  |
| **Income level** |  | 0.173 |  | 0.251 |  | 0.804 |
| Poor | 1.029(1.016,1.042) |  | 1.017(0.987,1.049) |  | 1.448(1.087,1.928) |  |
| Not poor | 1.038(1.029,1.048) |  | 1.058(0.996,1.123) |  | 1.400(1.156,1.697) |  |
| **Smoke** **status** |  | **0.004** |  | **0.01** |  | 0.712 |
| Never | 1.051(1.038,1.064) |  | 1.106(1.042,1.172) |  | 1.580(1.171,2.133) |  |
| Former | 1.036(1.023,1.048) |  | 1.008(0.991,1.026) |  | 1.339(1.102,1.627) |  |
| Now | 1.018(1.005, 1.032) |  | 1.056(1.006, 1.108) |  | 1.319(0.984, 1.768) |  |
| **Education attainment** |  | 0.095 |  | 0.526 |  | 0.65 |
| Less than 9th grade | 1.047(1.020,1.075) |  | 1.045(0.942,1.159) |  | 1.466(0.960,2.238) |  |
| 9-11th Grade | 1.026(1.008,1.046) |  | 1.079(1.018,1.144) |  | 1.388(0.914,2.110) |  |
| High school grad/GED | 1.031(1.018,1.044) |  | 1.015(0.962,1.071) |  | 1.545(1.123,2.124) |  |
| Some college or AA degree | 1.032(1.018,1.046) |  | 1.028(0.919,1.149) |  | 1.229(0.951,1.589) |  |
| College graduate or above | 1.060(1.037,1.083) |  | 1.110(1.019,1.209) |  | 1.592(1.012,2.503) |  |
| **Marital status** |  | 0.636 |  | 0.724 |  | 0.51 |
| Never married | 1.026(1.005, 1.048) |  | 1.070(0.992, 1.154) |  | 1.096(0.616, 1.947) |  |
| Married/Living with Partner | 1.035(1.024,1.045) |  | 1.029(0.955,1.108) |  | 1.432(1.137,1.802) |  |
| Widowed/Divorced/Separated | 1.039(1.027,1.052) |  | 1.065(1.007,1.127) |  | 1.436(1.116,1.846) |  |

**Supplementary Figure1. Receiver Operating Characteristic (ROC) Curve analysis of the relationship between different IR surrogates and Frailty**


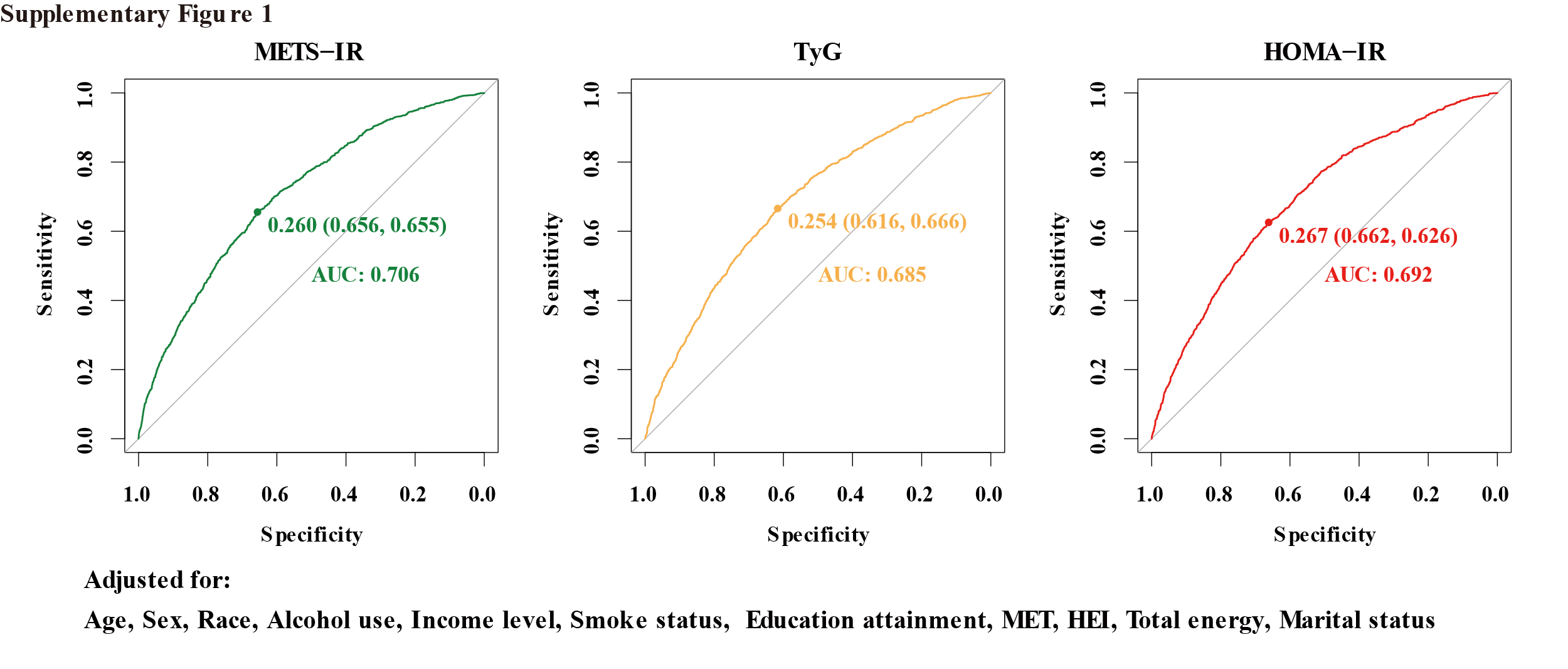

Supplement: Supplementary file 1 [file DataSheet1.docx]
